# Correction: The concept of “whole perforator system” in the lateral thoracic region for latissimus dorsi muscle-preserving large flaps: An anatomical study and case series

**DOI:** 10.1371/journal.pone.0314712

**Published:** 2024-11-26

**Authors:** Yu Kagaya, Masaki Arikawa, Takuya Sekiyama, Hideyuki Mitsuwa, Ryo Takanashi, Marie Taga, Satoshi Akazawa, Shimpei Miyamoto

In Table 1, there is an error in the total of Perforator Number. The correct total is: 4.51±1.44 (2~9). Please see the correct Table 1 here.

**Table 1:** A data summary of perforators on all the 175 sides.

	TDAP-sc	TDAP-mp	TDAP total (sc+mp)	LTAP	Total (TDAP+LTAP)
**Prevalence rate**	57.1% (100/175)	76.6% (134/175)	96.0% (168/175)	94.3% (165/175)	100.0% (175/175)
**Perforator number**	0.92±1.06 (0~5)	1.24±0.92 (0~4)	2.16±1.17 (0~7)	2.35±1.16 (0~6)	4.51±1.44 (2~9)
**Distance** **from the axillary artery**	148.7±56.3mm (8~272)	183.8±54.2mm (48~352)	168.8±73.1mm (8~352)	172.2±81.3mm (8~336)	170.6±71.0mm (8~352)
