# Supplementary figures and images for: The concept of “whole perforator system” in the lateral thoracic region for latissimus dorsi muscle-preserving large flaps: An anatomical study and case series
Source: PLoS One. 2021 Sep 2;16(9):e0256962. doi: 10.1371/journal.pone.0256962 (PMC8412279; doi:10.1371/journal.pone.0256962)

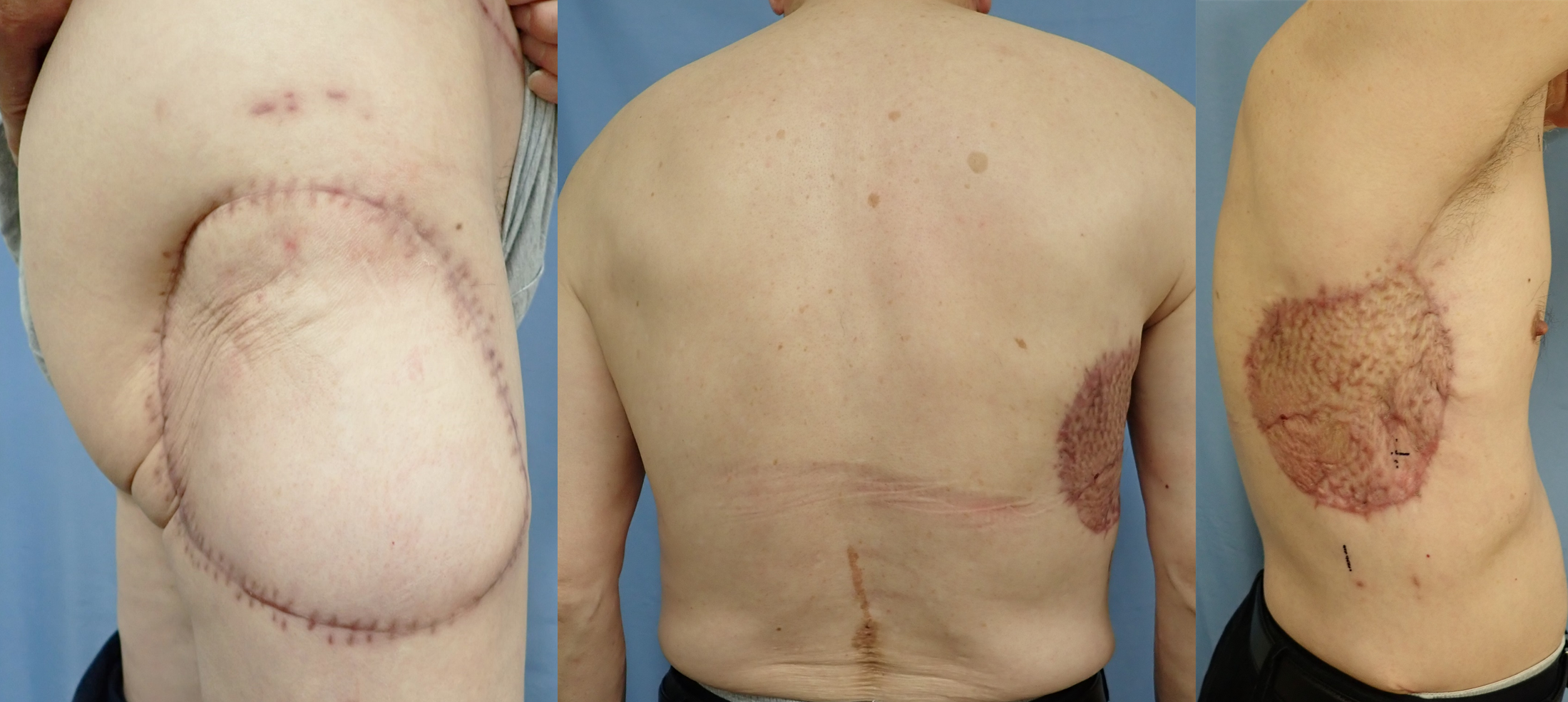

Supplement: S1 Fig — The flap survived completely. The donor-site of the split-thickness skin graft have marked pigmentation; however, there was no clinically problematic contracture or motility disturbance. (TIF) [file pone.0256962.s001.tif]

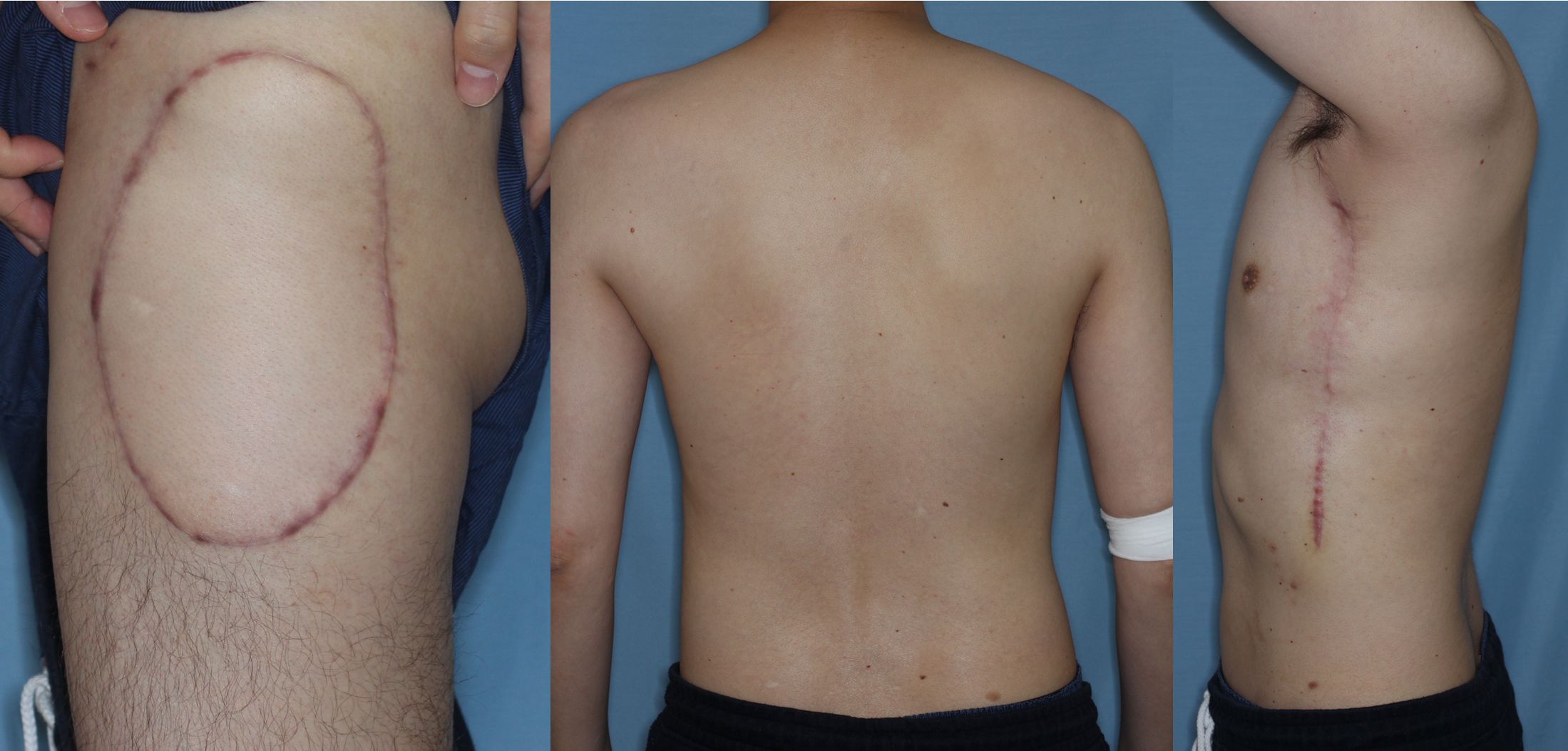

Supplement: S2 Fig — The flap survived completely. The donor-site of primary closure was invisible from the back and there was no clinically problematic contracture or motility disturbance. (TIF) [file pone.0256962.s002.tif]
