# Supplementary material for: The concept of “whole perforator system” in the lateral thoracic region for latissimus dorsi muscle-preserving large flaps: An anatomical study and case series
Source: PLoS One. 2021 Sep 2;16(9):e0256962. doi: 10.1371/journal.pone.0256962 (PMC8412279; doi:10.1371/journal.pone.0256962)
Supplement: Attachment — Submitted filename: CWPF MRI PLOS1 from Japan rv.docx [file pone.0256962.s003.docx]

**CWPF MRI PLOS1 from Japan rv**

**Introduction**

Whilst a hypothesis is stated, the introduction should state the aims of the study - not so much the methods. For example, we aimed, by analysing breast MRI scans to document the lateral chest wall perforator anatomy. Our secondary aim was to then apply findings from MRI to a patient cohort requiring large volume flaps with the lateral chest wall as a potential donor site.

Lines 81-2 – this really should be in methods – states:

“Moreover, the area of the LD lateral border is automatically included in the usual target range.”

Not sure this is the case – and certainly given standard imaging sequences for 1.5T may not be the case due to signal drop off around the LD m.

**Materials & Methods**

STROBE – is for prospective observational studies.

The MRI protocol does not mention patient positioning – in particular was the patient prone? What position were the arms in? were they both elevated above the patient’s head?

Regarding distance from the axillary artery (lines 110-111; “measured

111 as the distance from the axillary artery”) – was this estimated from the axillary artery to the ORIGIN of the perforator or to the most superficially visible part of the perforator?

Furthermore, can the authors clarify which images were used to document the perforator anatomy. Ie. What was the exact delay between contrast injection to sequence acquisition – ie the delay between GdDTPA injection and the images used?

Could the authors comment on whether the MRI was analysed by a radiologist or a surgeon or was there a second observer?

There is no mention of the surgical case series in the methods section. (lines 75-82 and 83-87 from introduction could go to the methods section).

There is also no mention of the sections used eg MPR - were only axial images analysed? Were MIPS constructed?

In a couple of the patient cases, ths use of color doppler is mentioned. Could the authors give a brief description of how they routinely assess each patient for suitable perforators in the clinic (pre-operatively)? Is color doppler done in the clinic? Is it done routinely intra-operatively or is hand-held doppler used?

**Results**

Given in lines 131-133 “Data of 156 consecutive patients were extracted in the predetermined period. Patients who did not undergo preoperative DCE-MRI or sides (right or left) that had images of unclear resolution due to artifacts or an incomplete target area due to a large body frame were excluded.”

And that only “175 sides of 98 patients were included”

Could the authors specify **exactly** how many patients **underwent MRI** and then of those patients **how many were evaluable**. This is important for readers so the % of MRI scans with evaluable information is understood.

From our experience analysing breast MRIs the more posterior TDAs/TDAPs may fall outside the window for accurate evaluation or else signal drop off occurs. Was this a significant reason for patients not to be included?

Excluding scans in patients with higher BMI / body frame may lead to a potential selection bias. Could the authors clarify if there was a cut-off for BMI? Or was it just based on scan / contrast quality/ sequence-contrast / image acquisition timing?

The authors stated that they ‘ignored’ small or unclear perforators – did they set a threshold for minimal diameter or signal?

Line 135 states “93 sides were diseased (breast cancer)” could the authors specify what the indication for MRI in other 5 patients (out of 98) were? (since they did not have any breast cancer).

Line 137 states “TDAP-sc existed” - I think rather than the word existed (implying that there was actually only what was seen on MRI – not substantiated by anatomical exploration), the authors should use the word “seen” so “TDAP-sc seen” as we can only comment on what was seen on MRI versus not seen.

Regarding the patient series: were any of these patients imaged by pre-operative MRI of the lateral chest wall to map perforator anatomy?

If not, are the authors saying that MRI was useful to provide a theoretical / anatomical framework for the lateral chest wall perforator anatomy? If so – this could be stated.

To clarify, the authors do NOT recommend DCE MRI as part of pre-op planning for patients undergoing harvesting of large flaps from the lateral chest wall?

Could the above issue be clarified please?

It would seem that the rationale for the MRI series is to plan for individual patients, the best main source artery to base the flap on (either LTA / TDA or both) and then incorporate the WPS as part of the flap dissection USING the individual patient’s anatomical map of communicating vessels. The latter seems important to understand prior to raising the flap as the level (cranio-caudal) of the communicating vessels between the 2 systems can vary (from proximal to distal).

For example in the discussion (lines 264-66) “there is a high variance of perforators that nourish the skin of the lateral thoracic area between cases, so that TDAP-sc or LTAP, which are

not utilized for usual TDAP flaps, are indispensable for skin flap perfusion and TDAP-mp.” Suggests that due to the variation seen in the MRI series that patients should undergo pre-operative imaging – especially if the lateral chest wall tissue may be tight and difficult to close if both in primary fashion if both anterior (LTAP) and posterior (TDAP) systems are harvested routinely? It then states that the anterior incision should be located “far anterior” to the LD muscle. But what about in females where the breast might be distorted laterally (as the authors later state).

**Table 1**

Number (xx~yy) seems incorrect for some values:

(2~94) under total perforator number per patient – **94**??? Is this range? Should it read 9?

Perforator Number 0.92**±1.06 – is the +/- range / SD / IQR? Please define in legend / table heading**

**DISTANCE from axillary artery:**

148.7±56.3mm – again is the +/- SD / IQR / range?

Again distance from axillary artery (8~**352**) – this implies distance from axillary artery >30cm???

Is this correct? Is it the range in (xx~yy)?

Given the variable distance of the perforator from the axillary artery, the real questions is what is the distance between the 3 perforators which make up the “whole perforator system (WPS)”? Was this generally within a certain distance? This would be a very relevant number clinically. **Could this line be added to the table?**

**Lines 151 – 153 states “**TDAP-sc was revealed to be significantly proximal to the TDAP-mp and the LTAP (both P<0.01), although there was no significant difference in the location between TDAP-mp and LTAP.”

This statement refers to the measurement comparing groups of patients.

It would be more useful to know for EACH individual patient, the distance between the perforators (origin?) - for clinical utility.

Lines 154 – 159 discuss confluence between main vessels or perforators. This is very interesting.

It would be useful to know left / right concordance for this aspect

**In Figures 3 –** it would be helpful to know how far distant to the axillary artery the examples of communications occurred. (in each case).

To what extent did pre-op MRI feature in planning the flap in the 7 clinical cases? This is unclear

**Case 2 fig 4 –** was TDA taken distal to transverse branch?

In figures 4A and 5A only the dominant perforator is shown (TDAPsc in 4A and TDAPmp in 5A). Were further perforators seen on color flow doppler / heard on acoustic doppler? If yes, why weren’t they marked? The description of the WPS is very detailed but the markings do not seen to reflect this.

Was the TDAPmp in 5A within 3cm of the LD lateral border? Why didn’t the authors mark communications between the LTA(P) system and the TD system? Were they looked for pre-op? Were they found?

In figure 4A – how do the authors know that the dominant perforator marked was a TDAP sc and NOT a LICAP? Was MRI done or careful color doppler mapping?

Case 2: How was the flap tip necrosis handled? What was the outcome?
